# Blocking Autophagic Flux Enhances Matrine-Induced Apoptosis in Human Hepatoma Cells

**DOI:** 10.3390/ijms141223212

**Published:** 2013-11-25

**Authors:** Li Wang, Chun Gao, Shukun Yao, Bushan Xie

**Affiliations:** 1 China-Japan Friendship Clinical Medicine College, Peking University Health Science Center, No.2 Yinghua East Road, Beijing 100029, China; E-Mails: liwang2011@bjmu.edu.cn (L.W.); xiebushan@gmail.com (B.X.); 2 Department of Gastroenterology, China-Japan Friendship Hospital, Ministry of Health, No.2 Yinghua East Road, Beijing 100029, China; E-Mail: gaochun@bjmu.edu.cn

**Keywords:** matrine, hepatocellular carcinoma, autophagy, apoptosis, autophagic inhibitor, chloroquine

## Abstract

Autophagy, a self-defense mechanism, has been found to be associated with drug resistance in hepatocellular carcinoma (HCC). Our study was designed to investigate the role and related mechanisms of autophagy in matrine-induced apoptosis in hepatoma cells of HepG2 and Bel_7402_. Cell apoptosis was detected by flow cytometry analysis (Annexin V–FITC/PI double-staining assay), the activity and activating cleavages of caspase-3, -8, and -9. MTT assay and colony forming assay were used to assess the effect of matrine on growth and proliferation of HCC cells. Autophagic flux in HCC cells was analyzed using the expression of LC3BI/II and p62/SQSTM1, GFP-LC3 transfection, and transmission electron microscopy. Moreover, regarding to the associated mechanisms, the effects of matrine on the phosphoinositide 3-kinase/AKT/mTOR pathway and beclin-1 were studied. Our results showed that: (1) both autophagy and apoptosis could be induced by treatment with matrine; (2) using the autophagic inhibitor chloroquine and beclin-1 small-interfering RNA, cell apoptosis induced by matrine could be enhanced in a caspase-dependent manner; and (3) autophagy was induced via inhibition of PI3K/AKT/mTOR pathway and up-regulation of beclin-1. In conclusion, inhibition of autophagy could enhance matrine-induced apoptosis in human hepatoma cells.

## 1. Introduction

Hepatocellular carcinoma (HCC) is one of the most common malignant neoplasms and a leading cause of cancer-related mortality in China [1]. Current major treatment modalities for HCC include surgical resection, liver transplantation, and local ablation therapies [2,3]. However, these treatments could not be used for many patients who were diagnosed with HCC at an advanced stage [4,5]. Unfortunately, very limited efficacy was shown for most of the conventional cytotoxic chemotherapeutic drugs in HCC therapy [6,7]. Some studies found that in HCC, the innate drug resistance, which is not fully understood, may result in a low response by HCC to most chemotherapies [7].

Matrine is an active component monomer that is extracted from *Sophora flavescens*, which is usually used as an anti-hepatic fibrosis drug [8]. Recent research has shown that matrine has anti-tumor effects on diverse tumor cell types, including HCC, pancreatic cancer, gastric cancer, and breast cancer [9–13]. Consistent with these published literature [14], our previous study found that both autophagy and apoptosis are increased in hepatoma cells exposed to matrine.

Normally, cells exhibit basal levels of autophagy as a conservative mechanism to perform homeostatic functions [15]. However, the intracellular response of autophagy is a double-edged sword. Recent studies found that for some types of tumors, the pro-survival effect of autophagy may contribute to drug resistance in chemotherapy [10,16,17]. The effect of autophagy depends on the type of tumor, and its role in HCC therapy has not yet been clarified. Moreover, autophagy is an intricate sequential process and studies have revealed that the entire process of autophagy, which is termed autophagic flux, usually, includes: a double-membrane phagophore is formed, expands around a portion of cytoplasm, and fuses to form the autophagosome, following which the autophagosomes fuse with the lysosomes to generate the autolysosomes [18].

Our study was designed to determine autophagic flux in matrine-treated hepatoma cells and revealed the association of matrine-induced autophagy with PI3K/AKT/mTOR pathway and beclin-1. Furthermore, we would study the matrine-induced apoptosis in hepatoma cells, during inhibition of autophagic flux, using chemical inhibition or depletion of autophagy-related gene (*Atg*).

## 2. Results

### 2.1. Matrine Inhibited Viability of HepG2 and Bel_7402_ Cells

**Figure 1.**
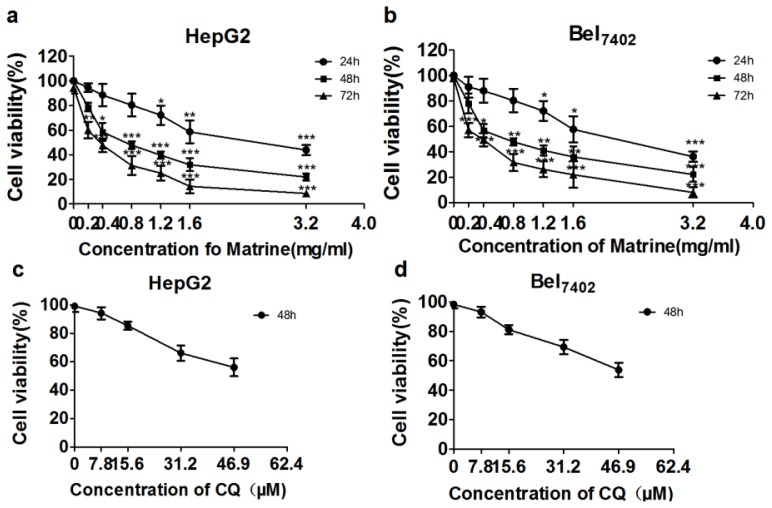
MTT assay showing the cytotoxicity of matrine or CQ on HCC cells. (**a**) HepG2 and (**b**) Bel_7402_ cells were treated with matrine at concentrations of 0.2, 0.4, 0.8, 1.2, 1.6, and 3.2 mg/mL for 24, 48, and 72 h. Matrine inhibited the growth of HepG2 and Bel_7402_ cells in a dose- and time-dependent manner. The optimal concentration was determined by the half maximal inhibitory concentration calculated using GraphPad software; (**c**) HepG2 and (**d**) Bel_7402_ cells were also treated with CQ at concentrations of 7.8, 15.6, 31.2, and 46.9 μM for 48 h. Data are means ± SD of three independent experiments. *p < 0.05, **p < 0.01,***p < 0.001 *vs*. control group.

The inhibitory effect of matrine on HepG2 and Bel_7402_ cells was detected by MTT assay. The results showed that matrine inhibited the viability of HepG2 and Bel_7402_ cells in a dose- and time-dependent manner after the cells were treated with matrine at 0.2–3.2 mg/mL for 24, 48, and 72 h (Figure 1). The cells displayed significantly reduced viability, especially at 48–72 h. According to the MTT assay results, the half maximal inhibitory concentration values of matrine treatment for 48 h were 0.8 ± 0.03 mg/mL and 0.8 ± 0.09 mg/mL in HepG2 and Bel_7402_ cells, respectively. Thus, 0.8 mg/mL of matrine was used as the working concentration and 48 h was used as the incubation time in subsequent experiments. Chloroquine (CQ) has been reported to have cytotoxic potential [19]. To confirm that the synergistic effect of matrine and CQ is due to the impairment of autophagy and not to the cytotoxicity of chloroquine itself, we detected the cytotoxicity of chloroquine at concentrations of 7.8–46.9 μM for 48 h and used a concentration of 7.8 μM CQ, which displayed apparent non-cytotoxicity to cells (Figure 1), in further experiments.

### 2.2. CQ Enhanced Suppressive Effect of Matrine on Proliferation of Hepatoma Cells

**Figure 2.**
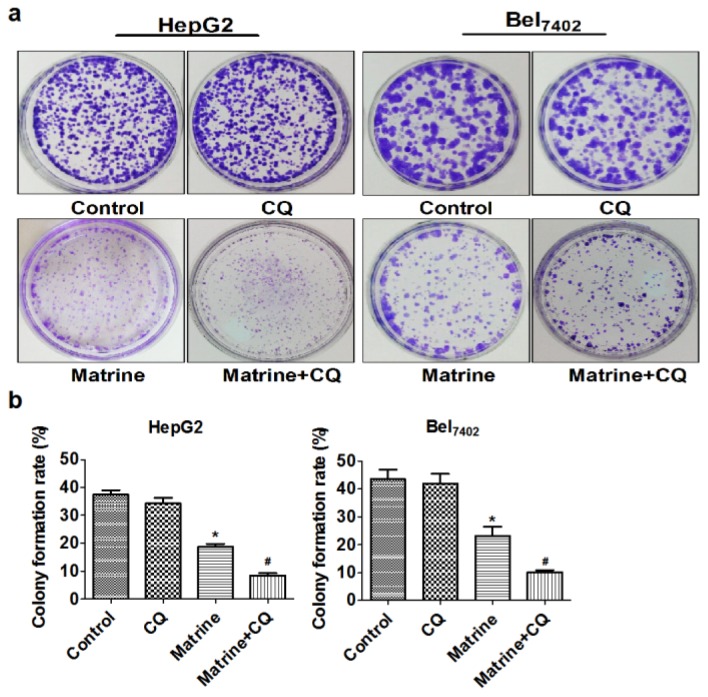
The suppressive effect of matrine with or without CQ on colony formation of hepG2 and Bel_7402_ cells. (**a**) Representative images of colony-forming assay; (**b**) Analysis of Colony formation rates of hepG2 and Bel_7402_ cells. Data are means ± SD of three independent experiments. *p < 0.05 *vs*. control group, ^#^*p* < 0.05 *vs*. matrine group.

Colony formation assay was done to investigate long-term effect of matrine on proliferation of hepG2 and Bel_7402_ cells. As shown in Figure 2, the colony-forming ability of both cell lines was reduced after exposure to matrine. Furthermore, the reduction was even more remarkable after matrine was combined with CQ. The results indicated that CQ contributed to the suppressive effect of matrine on proliferation of hepG2 and Bel_7402_ cells.

### 2.3. CQ Blocked Matrine-Induced Autophagic Flux in Hepatoma Cells

In recent years, autophagic flux has been widely used to denote the dynamic process of autophagy. Monitoring autophagic flux provides a more accurate way to detect the true level of autophagy.

**Figure 3.**
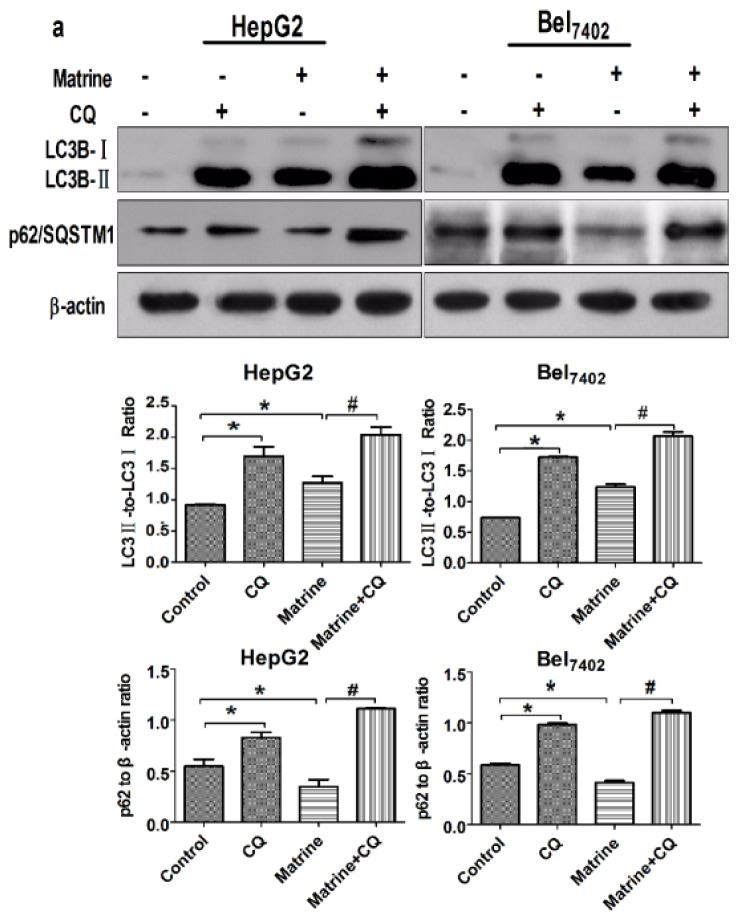

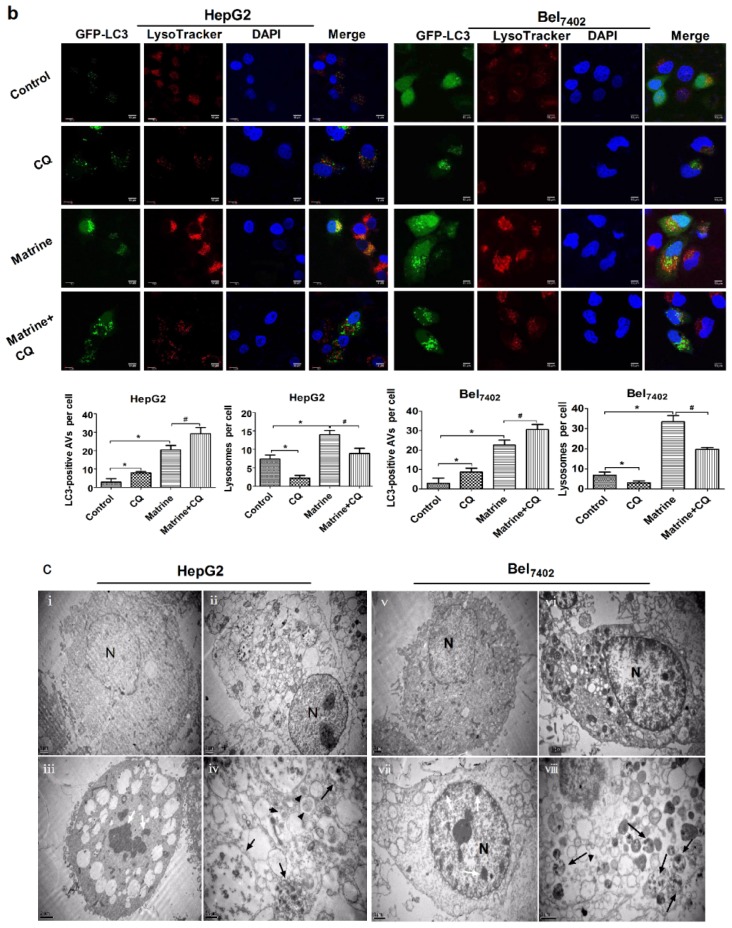
CQ inhibited matrine-induced autophagic flux in HepG2 and Bel_7402_ cells. HepG2 and Bel_7402_ cells were treated with 0.8 mg/mL matrine in the absence or presence of CQ (7.8 μM) for 48 h. (**a**) Western blot analysis of expression of the LC3BII/LC3BI ratio and p62/SQSTM1. β-actin is shown as a loading control; (**b**) Representative confocal images after transfection with GFP-LC3 and staining with LysoTracker Red. The positive GFP-LC3 puncta represent the formation of autophagosomes. The quantification of LC3-positive autophagosomes and lysosomes labeled with LysoTracker Red in HepG2 and Bel_7402_ cells were performed using Image J. Nuclei were stained with DAPI (blue). Scale bars, 10 μm; (**c**) Representative transmission electron micrograph of HepG2 and Bel_7402_ cells treated with matrine (0.8 mg/mL for 48 h): (**i**,**v**) Control cells without matrine; (**ii**–**iv**,**vi**–**viii**) Cells treated with matrine; Abundant autophagic vacuoles were observed after treatment with matrine in (**ii**) and (**vi**); Typical autophagic vacuoles are magnified in (**iv**) and (**viii**); Autophagosomes (early autophagic compartments; black arrowheads) are double-membrane structures that engulf cellular contents, while autolysosomes (late autophagic compartments; black arrows) are single-membrane structures containing degraded cellular contents. Typical apoptotic changes such as chromatin condensation, nuclear fragmentation, and apoptotic bodies are indicated with white arrows in (**iii**) and (**vii**). N, nucleus. Scale bars, 1 μm (**i**–**iii**, **v**–**vii**) and 0.5 μm (**iv**, **viii**). Data are means ± SD of three independent experiments. *p < 0.05 *vs*. control group, ^#^*p* < 0.05 *vs*. matrine group.

Autophagic flux in HepG2 and Bel_7402_ cells was monitored after treatment with matrine, in the presence or absence of CQ. The influence of matrine, with or without CQ, on LC3 (microtubule-associated protein 1 light chain 3)-II levels and p62/sequestosome 1 (SQSTM1) was assessed by Western blot. The LC3 proteins, which are involved in the formation of autophagosomes, generally serve as autophagic markers [20]. P62/SQSTM1, as a substrate of autophagy, can be degraded by autolysosomes. Autophagy deficiency can lead to the accumulation of p62/SQSTM1 [21]. As shown in Figure 3a, matrine induced the lipidation of LC3I to LC3II and degradation of p62/SQSTM1. The addition of CQ further increased LC3BII and p62/SQSTM1 accumulation, compared with cells treated with matrine only.

Next, we used GFP-LC3 plasmids and LysoTracker Red to monitor the efficiency of autophagosome/lysosome fusion in autophagic flux. LysoTracker Red is an acidophilic, lysosomotropic dye that labels the acidic lysosome. After treatment with drugs, we observed numerous GFP-LC3 puncta and dot-like red fluorescence accumulated in the cytosol in the matrine group compared with the control group, while the matrine plus CQ group showed more GFP-LC3 puncta but less red fluorescence (Figure 3b). The co-localization of GFP-LC3 with LysoTracker Red was presumably due to the fusion of autophagosomes and lysosomes, while the reduction in co-localization after the addition of CQ suggests impairment of the fusion process (Figure 3b). These findings demonstrated that matrine induced autophagic flux in hepatoma cells, and CQ arrested autophagic flux by impairing the fusion of autophagosomes and lysosomes into autolysosomes.

Furthermore, a study of ultrastructural morphology by transmission electron microscopy further confirmed the presence of abundant autophagic vacuoles sequestrating the cytoplasm and organelles in the matrine group. Both, autophagosomes with typical double membranes, and autolysosomes with degraded remnants, were observed (Figure 3c). In addition, the typical morphologies of apoptosis, such as cytoplasmic vacuolation, chromatin condensation, and nuclear fragmentation were also observed in HepG2 and Bel_7402_ cells in the matrine group. Moreover, as shown in Figure 3c, the cells with more typical apoptotic nuclear morphological changes displayed fewer autophagic vacuoles in the cytoplasm, suggesting that autophagy is a cellular survival response of HepG2 and Bel_7402_ hepatoma cells under toxic stress.

### 2.4. Combined Treatment with the Autophagic Inhibitor CQ Enhanced Matrine-Induced Apoptosis in a Caspase-Dependent Manner

**Figure 4.**
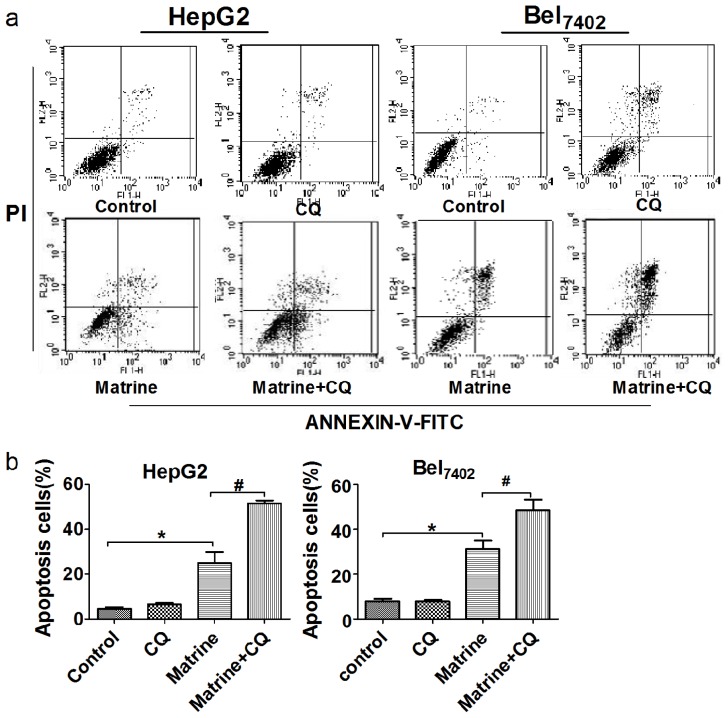

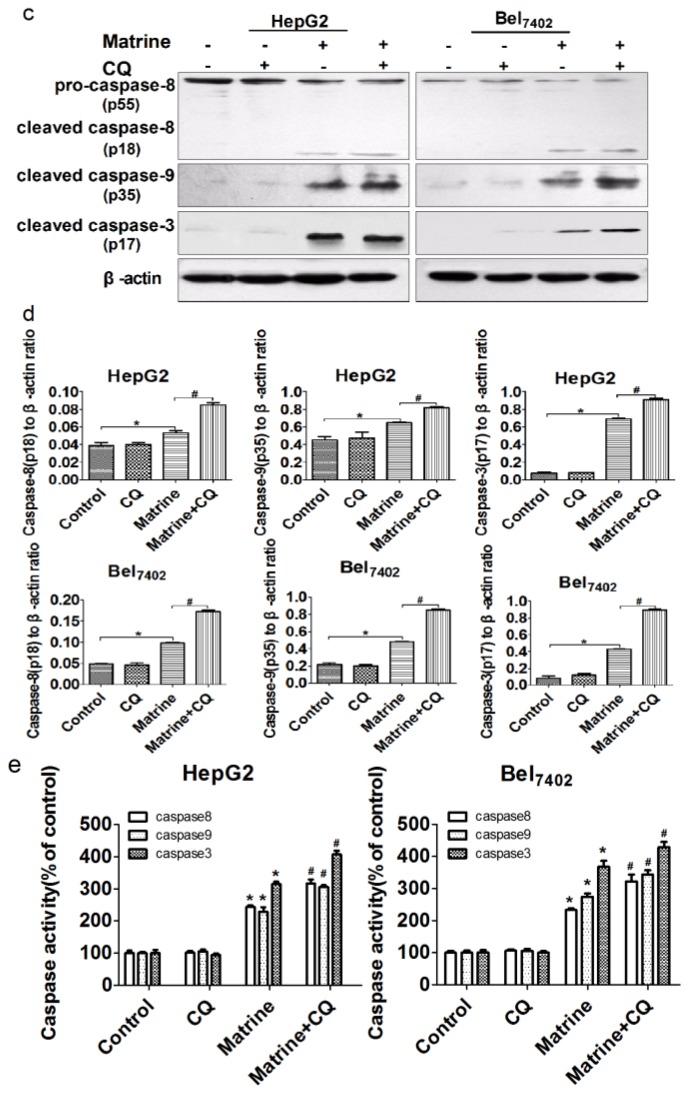
Combined treatment with the autophagic inhibitor CQ promoted matrine-induced apoptosis. (**a**) HepG2 and Bel_7402_ cells were treated with matrine 0.8 mg/mL in the absence or presence of CQ (7.8 μM) for 48 h. Annexin V-FITC and PI double-staining shows the percentages of early (bottom right) and late (top right) apoptotic cells; (**b**) Analysis of the total percentage of early and late apoptotic cells in different groups; (**c**) Western blot analysis of activated caspase-3, -8, and -9 expression in HepG2 and Bel_7402_ cells treated with 0.8 mg/mL matrine in the absence or presence of CQ (7.8 μM) for 48 h. β-Actin is shown as a loading control; (**d**) Analysis of relative expression of activated caspase-3, -8, and -9; (**e**) Activity of caspase-3, -8, -9 in HepG2 and Bel_7402_ cells after treated with matrine 0.8 mg/mL in the absence or presence of CQ. Data are from three independent experiments. *p < 0.05 *vs*. control group, ^#^*p* < 0.05 *vs*. matrine-treated group.

Cellular apoptosis was detected by annexin V-fluorescein isothiocyanate (FITC)/propidium iodide (PI) double-staining. Flow cytometry analysis showed that the total apoptosis rates of HepG2, including early (annexin V+/PI−) and late (annexin V+/PI+) stages, were 4.48% ± 1.43% in the control group, 6.68% ± 0.88% in the CQ group, 24.85% ± 8.79% in the matrine-treated group, and 54.92% ± 3.68% in the matrine plus CQ-treated group; the total apoptosis rates of Bel_7402_ were 8.01% ± 1.88% in the control group, 7.88% ± 1.09% in the CQ group, 31.18% ± 6.4% in the matrine-treated group, and 48.35% ± 8.42% in the matrine plus CQ-treated group. A 7.8 μM dose of CQ induced little apoptosis of HepG2 and Bel_7402_ cells, but the addition of CQ augmented matrine-induced apoptosis, compared with matrine alone (Figure 4a,b). Similar effects were further confirmed by evaluation of the cellular activity of caspase-3, -8, -9 by colorimetric assay and the cleavage of caspase-3, -8, and -9 by Western blot. The results showed that caspase-3, -8, and -9 were activated after incubation with matrine, compared with control groups. Moreover, higher levels of activated caspases were detected on combined incubation with the autophagic inhibitor CQ than in the matrine-only group (Figure 4c–e). These results demonstrated that the addition of CQ can promote matrine-induced apoptosis of hepatoma cells in a caspase-dependent manner.

Collectively, the results indicated that matrine-induced autophagy may constitute a pro-survival compensatory mechanism, as inhibition of autophagy drove more cells to die of apoptosis.

### 2.5. Matrine-Induced Autophagy via Inhibition of the PI3K/AKT/mTOR Pathway and Up-Regulation of Beclin-1

**Figure 5.**
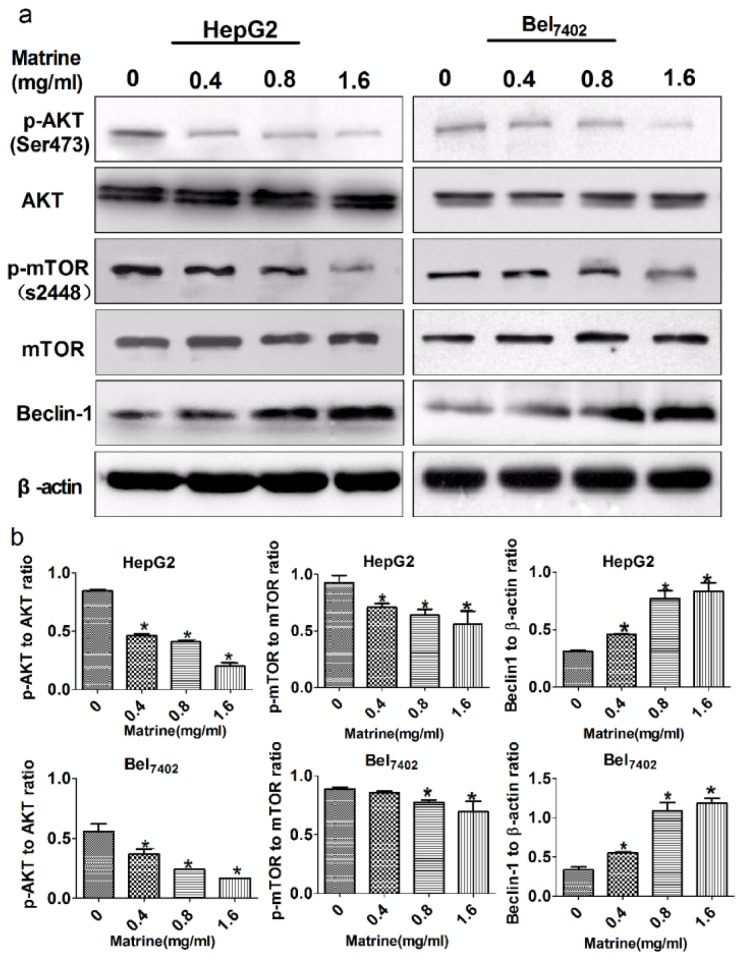
PI3K/AKT/mTOR signaling and beclin-1 are involved in matrine-induced autophagy. (**a**) Western blot analysis of pAKT, total AKT, p-mTOR, total mTOR, and beclin-1 in HepG2 and Bel_7402_ cells. β-actin is shown as a loading control. Data are from three independent experiments; (**b**) Analysis of relative expression of pAKT, total AKT, p-mTOR, total mTOR, and beclin-1. *p < 0.05 *vs*. control group.

To investigate the mechanism of matrine-induced autophagy, we looked at the kinasemammalian target of rapamycin (mTOR), and inhibition of mTOR was revealed as a key target in triggering autophagy [22]. After exposure to matrine, expression of mTOR and phosphorylated mTOR (p-mTOR) at Ser2448 was detected by Western blot. As shown in Figure 4, p-mTOR was reduced in a dose-dependent manner, while the expression of total mTOR did not change markedly. AKT is the main upstream target gene of mTOR, and positively regulates the activity of mTOR by phosphorylation. Here, we also detected the effect of matrine on AKT and pAKT at Ser473: the expression of pAKT was downregulated, without obvious changes in the total AKT. Our results demonstrated that inactivation of the phosphoinositide 3-kinase (PI3K)/AKT/mTOR pathway may be involved in matrine-induced autophagy. The process of autophagy is tightly regulated by autophagy-related-genes (ATGs). We also detected expression of beclin-1, the mammalian ortholog of ATG6, a key autophagy inducer, which could regulate autophagy positively in an mTOR-independent manner [23]. Western blot showed that beclin-1 levels in HepG2 and Bel_7402_ cells were upregulated after exposure to matrine (Figure 5). Accordingly, matrine-induced autophagy was mediated both by inactivating PI3K/AKT/mTOR pathway and up-regulation of beclin-1.

### 2.6. Silencing Beclin-1 Promoted Matrine-Induced Apoptotic Cell Death through Blockage of Autophagic Flux

**Figure 6.**
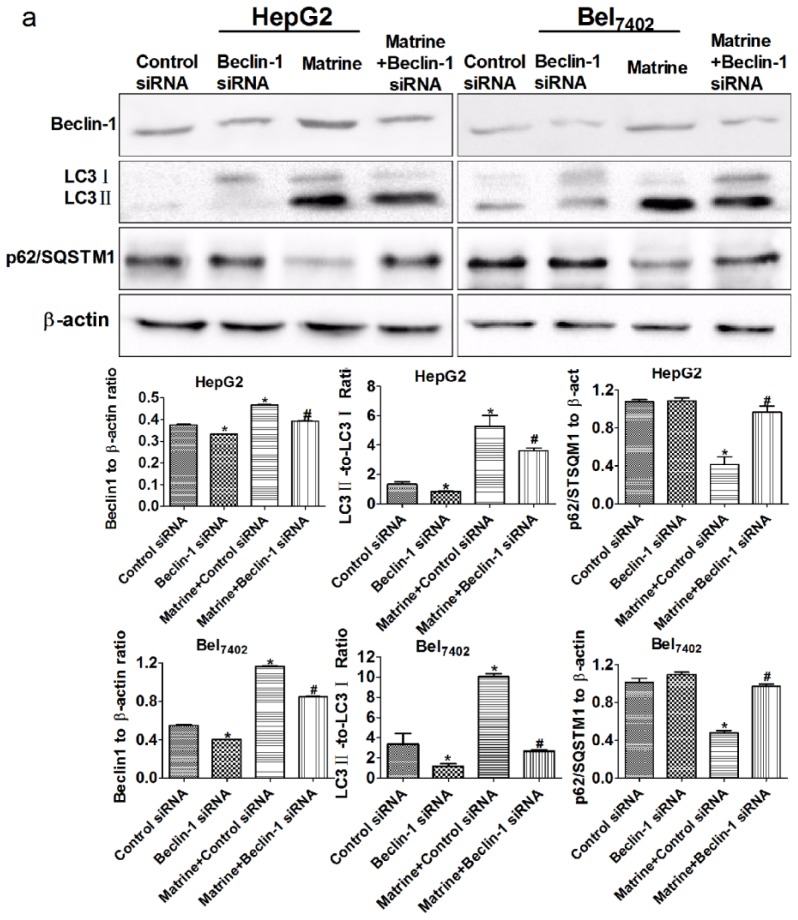

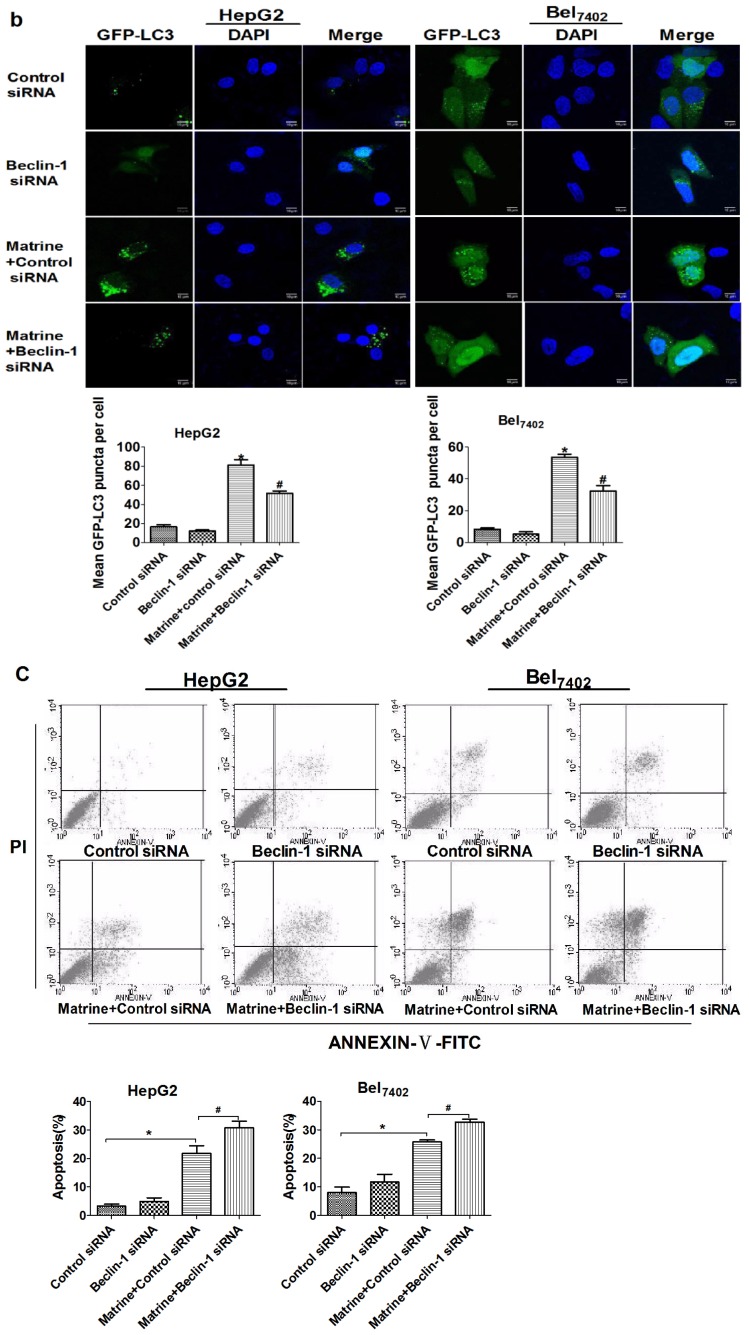
Effects of beclin-1 knockdown on matrine-induced autophagic flux and apoptosis in HepG2 and Bel_7402_ cells. (**a**) Beclin-1 expression, the LC3BII/LC3BI ratio and p62/SQSTM1 in HepG2 and Bel_7402_ cells after transfection with beclin-1 siRNA were detected by western blot. β-actin is shown as a loading control; (**b**) Representative confocal images of beclin-1-deficient cells after transfection with GFP-LC3 and treatment with matrine. LC3-positive autophagosomes in HCC cells were quantified using Image J (1.42q, NIH, Bethesda, MD, USA, 2009). Scale bars, 10 μm; (**c**) Apoptosis of HepG2 and Bel_7402_ cells transfected with beclin-1 siRNA after treatment with matrine was detected by Annexin V-FITC and PI double-staining. Apoptotic cell death was analyzed by the total percentage of early and late apoptotic cells in different groups. Data are means ± SD of three independent experiments. *p < 0.05 *vs*. control group, ^#^*p* < 0.05 *vs*. matrine group.

To further investigate the effect of impairing autophagy on matrine-induced apoptosis, we silenced beclin-1 (ATG6) using small-interfering RNA (siRNA). The depletion of beclin-1 was detected by western blot. The expression of beclin-1 in HepG2 and Bel_7402_ cells was clearly attenuated after transfection with specific siRNA, compared with control siRNA or the matrine group (Figure 6a). We then assessed changes in autophagic flux in HCC cells using the LC3BII/LC3BI ratio and p62/SQSTM1 by Western blot and GFP-LC3 transfection. As shown in Figure 6a,b, the reduction in LC3II and GFP-LC3 puncta, together with the accumulation of p62/SQSTM1 in beclin-1 siRNA-transfected groups, revealed down-regulation of autophagic flux. Collectively, depletion of beclin-1 could inhibit the formation of autophagosomes to block matrine-induced autophagic flux at an early stage in HCC cells. Subsequently, we analyzed matrine-induced apoptosis of beclin-1-deficient cells by flow cytometry. Apoptotic cell death, after exposure to matrine in the beclin-1 siRNA-transfected group, showed an obvious increase in both HepG2 and Bel_7402_ cells, compared with the control siRNA-transfected group (Figure 6c). These results indicate that beclin-1 is a key inducer of matrine-induced autophagy, and inhibition of autophagy via depletion of beclin-1 may promote matrine-induced apoptosis. These results are consistent with our previous study of blocking autophagic flux with the pharmaceutical autophagic inhibitor CQ.

## 3. Discussion

Our study found that, matrine induced both autophagy and apoptosis in hepatoma cells, and inhibition of autophagy enhanced matrine-induced apoptosis, which is related to the activity of caspase-8, -9, -3. Caspases, types of cysteine proteases closely linked to apoptotic cell death, normally exist as inactive forms. When apoptosis is initiated, caspases are cleaved to be active enzymes. Caspase-8, -9, -3 are the important junctions of apoptosis pathways. Caspase-8, the initiator caspase, once activated by the apoptotic stimuli, on one side triggers the release of cytochrome c from mitochondria via cleavage of BID or directly activates the downstream caspase by proteloytic cleavage [24]. Released cytochrome c could directly promote caspase-9 activation. Caspase-3 is the downstream effector caspase, which could amplify the initiating signals of caspase-8, and -9 and caused apoptotic chromatin condensation and DNA fragmentation [25]. Consistent with our results, some frontline chemotherapeutic drugs have been found to induce autophagy, for example sorafenib, etoposide, and 5-fluorouracil [26–28]. However, the role of autophagy in chemotherapy remains controversial. Under some circumstances, autophagy is an adaptive mechanism, which can sustain cell survival under stressful conditions. In contrast, it has been suggested that excessive autophagy ultimately induces non-apoptotic programmed cell death, called “type II” or “autophagic cell death”. In our study, both autophagy and apoptosis were observed in matrine-treated human hepatoma cells. To clarify the role of autophagy, we study the cytotoxicity of matrine and matrine-induced cell death after inhibition of autopahgy. The results showed that inhibition of autophagy did not result in a decrease, but an enhancement of cell death as quantified by flow cytometry, leading to our suggesting that autophagy protects hepatoma cells from apoptosis under matrine treatment and probably plays a pro-survival role in marine-induced hepatoma cell injury. Similar findings were also reported in other tumor cells [10].

Moreover, we examined the expression of LC3II and p62/SQSTM1, substrates of autophagic flux, and the process of autophagosome-lysosome fusion. An increase of LC3 conversion without p62 expression indicates induction of autophagic flux, whereas the increase of LC3 conversion with p62 expression indicates the inhibition of autophagic flux [29]. The results then were further proved by transmission electron through directly observing the ultrastructural morphological features of autophagy and apoptosis. In our study, we found that CQ blocked the degradation of autolysosomes at a late stage of autophagy, whereas knockdown of beclin-1 inhibited the formation of autophagosomes at an early stage. The similar result was observed that cytotoxicity was enhanced by blocking autophagic flux, although these experiments targeted different stages of autophagic flux. However, our study was not supported by other studies, which reported that the cytotoxicity of chemotherapy agents is attenuated by inhibiting autophagy at an early stage, but augmented by inhibiting autophagy at a late stage [30]. Targeting stage-specific autophagic flux may affect the efficacy of modulating the cytotoxicity of drugs.

Many different autophagy inhibitors have been used, including wortmannin, 3-methyladenine, bafilomycin A1, Chloroquine, and hydroxychloroquine (HCQ). CQ and HCQ, mostly used as the anti-malarial and anti-inflammatory agents, have been found, in recent years, to have the new function for the inhibition of autophagy through the prevention of lysosome acidification. More than 20 clinical trials of CQ or HCQ combined with chemotherapeutic drugs are underway, and some of the preliminary data have shown that the combination of CQ or HCQ with chemotherapeutic drugs made tumors more sensitive to the chemotherapeutic drugs and prolonged survival times [31]. The anti-tumor mechanisms of CQ are not well understood. However, studies have revealed that arresting autophagy at a late step may contribute to the anti-tumor effect of CQ [32]. For the first time, our study demonstrated that a low-dose CQ could enhance matrine-induced apoptosis by blockage of autophagic flux, and put forward the potential possibility of combination of matrine and CQ in HCC therapy.

Our study also demonstrated that both beclin-1 and the PI3K/AKT/mTOR signaling pathway play some roles in matrine-induced autophagy. Beclin-1, the mammalian ortholog of Atg6, is a well-known autophagy regulator. Researches have revealed that beclin-1 is a tumor suppressor and that beclin-1 deficiency can promote the formation and transformation of liver cancer cells [33]. Moreover, beclin-1 is a key regulator that can regulate the crosstalk between autophagy and apoptosis [34]. Our study showed that depletion of beclin-1 can promote matrine-induced apoptosis. The PI3K/AKT/mTOR signaling pathway plays an important role in regulating cell growth and proliferation of normal and tumor cells. Over-activation of this pathway has been demonstrated to contribute to cancer proliferation and progression in various human malignancies. Moreover, aberrant activation of the PI3K/AKT/mTOR signaling pathway is frequently found in HCC and is associated with poor prognosis and high-grade HCC [35,36]. Considering potential therapeutic targets for malignancies, inhibitors targeting the key components of this pathway have been widely studied and some of them are undergoing clinical trials, such as AKT and mTOR [37]. Our study showed that matrine is a dual inhibitor of AKT and mTOR, which may contribute to the anti-HCC effect of matrine. In addition to HCC, the inhibitory effect of matrine on the PI3K/AKT/mTOR pathway has also been reported in gastric cancer, acute myeloid leukemia, breast cancer, and other malignancies [9,10,38]. In addition, inhibition of the PI3K/AKT/mTOR signaling pathway can result in the induction of autophagy [22]. Some studies have shown that inhibition of autophagy might overcome the therapeutic resistance to PI3K/AKT/mTOR signaling inhibitors in some types of tumors [39–41]. Similar results were also observed in our study, which showed that addition of the autophagy inhibitor CQ enhanced matrine-induced cell apoptosis. We hope that these results could be used in the therapy of HCC in future.

## 4. Experimental Section

### 4.1. Reagents and Antibodies

Matrine and CQ (purity > 99%) were purchased from National Institute for Food and Drug Control of China and dissolved with double-distilled H_2_O to make stock solutions of 10 and 20 mg/mL, respectively. Anti-LC3B antibody was purchased from Sigma-Aldrich (St. Louis, MO, USA). Antibodies against activated caspase-3, -8, and -9 were purchased from Epitomics (Burlingame, CA, USA). Antibodies against pAKT and AKT were purchased from Cell Signaling Technology (Danvers, MA, USA). Antibodies against p-mTOR and mTOR were purchased from Abcam (Cambridge, UK). Antibody against beclin-1, control siRNA, and beclin-1 siRNA were purchased from Santa Cruz Biotechnology (Santa Cruz, CA, USA). Antibody against β-actin was purchased from MBL Technologies (Woburn, MA, USA). Antibody against p62/SQSTM1 was purchased from Biosynthesis Biotechnology (Beijing, China). The annexin V-FITC/PI reagent kit was purchased from Beijing Biosea Biotechnology (Beijing, China). The MTT assay kit was purchased from Solarbio Biotechnology (Shanghai, China). Lipofectamine 2000 transfection reagent was purchased from Invitrogen (Carlsbad, CA, USA). GFP-LC3 plasmids were a kind gift from Dr. Xie BS from the Biochemistry Department of the Health Science Center of Peking University.

### 4.2. Cell Lines and Cell Culture

Human hepatoma cell lines HepG2 and Bel_7402_ were obtained from the Biochemistry Department of the Health Science Center of Peking University. HepG2 cells were routinely cultured in Dulbecco’s modified Eagle’s medium (Gibco BRL, Grand Island, NY, USA). Bel_7402_ cells were cultured in RPMI 1640 medium (Thermo Scientific HyClone, Beijing, China). DMEM and RPMI 1640 were both supplemented with 10% (*v*/*v*) fetal bovine serum (Gibco BRL, Grand Island, NY, USA). All cells were incubated at 37 °C in a humidified 5% CO_2_ atmosphere.

### 4.3. Plate Colony Forming Assay

Cells at the exponential growth phase were harvested with trypsin-EDTA and counted using a hemocytometer. Following this, cells were diluted and seeded at about 1000 cells per well of a six-well plate. After a 12-h incubation, cells were treated for 24 h with 0.8 mg/mL matrine in the absence or presence of CQ (7.8 μM), and then continuously incubated in new fresh medium at 37 °C in 5% humidified CO_2_. After incubation for 10–14 days, cells were washed with PBS twice, fixed with methanol for 15 min, and stained with 0.5% crystal violet for 15 min at room temperature. The colony is defined to consist of at least 50 cells. Visible colonies were counted. Colony formation rate = (number of colonies/number of seeded cells) × 100%.

### 4.4. Measurement of Cell Viability

Viability of cells was assessed by MTT assay. The cells were seeded in 96-well flat-bottom microtiter plates (Corning Inc., Corning, NY, USA) at a density of 5 × 10^3^ cells per well, allowed to adhere overnight, and then treated with matrine at concentrations of 0.2, 0.4, 0.8, 1.2, 1.6, and 3.2 mg/mL for 24, 48, and 72 h. A control-group and zero-adjustment well were also set. An MTT solution (5 mg/mL) was added at the end of incubation, and incubation was continued for 4 h. The reaction was terminated by adding a detergent reagent. The absorbance value in 96-well plate was read spectrophotometrically at 570 nm on a microtiter plate reader (Thermo Scientific, West Palm Beach, FL, USA). An MTT assay for CQ at concentrations of 7.8, 15.6, 31.2, and 46.9 μM for 48 h were performed as described above. All MTT assays were performed in triplicate. The inhibitory rate for the proliferation of HCC cells was calculated according to the formula: (1 − experimental absorbance value/ control absorbance value) × 100.

### 4.5. Flow Cytometry Analysis of Apoptosis

Following drug treatment, cells were harvested by treating with trypsin. Total cells were then twice washed with cold phosphate-buffered saline (PBS) and resuspended in the annexin V binding buffer at a concentration of 1 × 10^6^ cells/mL. A single-cell suspension (100 μL) was stained with 5 μL annexin V-FITC for 15 min at room temperature in the dark, and then incubated with PI for 2 min. After the incubation period, 400 μL of annexin V binding buffer was added to each tube, followed by flow cytometry (FACSAria, BD Biosciences, San Jose, CA, USA). The extent of apoptosis was quantified as a percentage of annexin V-FITC positive cells.

### 4.6. Caspase Activity

The activity of Caspase-3, -8, and -9 were performed separately by using colorimetric activity assay kits (KeyGEN Biotech, Nanjing, China). According to the instructions, (3 × 10^6^)–(5 × 10^6^) cells were lysed in a lysis buffer containing 1% DL-Dithiothreito (DTT) on ice for 60 min and centrifuged at 10,000× *g* for 15 min. The protein concentration was determined using BCA protein assay. To detect the activity of aspase-3, -8, and -9, assays were performed by incubating 200 μg protein of cell lysate from different group in 100 μL of reaction buffer containing 5 μL of caspase-3, -8, and -9 substrate and 1% DTT in 96-well plates at 37 °C, avoiding light for 4 h. The activity of caspase-3, -8, and -9 were evaluated using a spectrophotometer at 405 nm.

### 4.7. Cell Lysis and Western Blot Analysis

Cells lysates were prepared by extracting proteins with RIPA lysis buffer supplemented with proteinase inhibitors. The protein samples were detected by SDS-polyacrylamide gel electrophoresis (PAGE). About 30 μg of protein was loaded onto each lane of SDS-PAGE gel, and transferred onto a nitrocellulose membrane. After blocking with 5% non-fat milk in Tris-buffered saline containing 0.1% Tween-20, the membranes were probed with designated first and second antibodies. The immunoreactive signals were stained using a chemiluminescent reagent kit (Millipore, Schwalbach, Germany). Chemical illuminances were detected using a cooled CCD camera (Bio-Rad, Foster, CA, USA). Western blotting images were measured using Image J software (1.42q, NIH, Bethesda, MD, USA, 2009).

### 4.8. GFP-LC3 Plasmid Transfection

Cells were seeded onto six-well plates and transfected with a GFP-LC3 expression plasmid at approximately 60%–70% confluence using the Lipofectamine 2000 transfection reagent (Invitrogen, Carlsbad, CA, USA). After 48 h, the cells were treated with matrine with or without CQ. For observation, cells were fixed with 4% formaldehyde for 15 min, and then washed twice in cold PBS. Cell nuclei were counterstained with DAPI. Cells were imaged from four non-overlapping fields under an Olympus FV500 confocal laser-scanning microscope. GFP-LC3-positive puncta were counted using Image J software (1.42q, NIH, Bethesda, MD, USA, 2009).

### 4.9. Transmission Electron Microscopy

Cells were fixed in 3% glutaraldehyde in 0.1 M sodium cacodylate buffer, pH 7.4, at 4 °C overnight and postfixed with 1% osmium tetroxide in 0.1 M sodium cacodylate buffer, pH 7.4, for 1.5 h at room temperature. After fixation, cells were dehydrated in a gradient of 50%–100% ethanol and embedded in SPI-Pon 812 resin. Ultrathin sections (70 nm) were obtained using a microtome (Leica Ultracut; Leica Microsystems, Wetzlar, Germany). After staining with lead citrate/uranyl acetate, the sections were examined under a transmission electron microscope (JEM-1400; JEOL, Tokyo, Japan) at 80 kV.

### 4.10. siRNA Transfection

Cells were seeded at approximately 2 × 10^5^ cells per well onto a six-well tissue culture plate. When the cells were 60%–70% confluent, the cells were transfected with control siRNA and target-gene siRNA with the Lipofectamine 2000 reagent. The cells were then incubated with or without matrine for further experiments, and the effect of target-gene knockdown was verified by Western blot.

### 4.11. Statistical Analysis

All data are expressed as means ± standard deviation (SD). Statistical analysis was performed using Prism 5 (GraphPad, San Diego, CA, USA, 2007) and *p* ≤ 0.05 was considered statistically significant. The data were analyzed by analysis of variance followed by Student’s t-test for multiple comparisons.

## 5. Conclusions

In summary, our results showed that matrine could induce apoptosis and autophagy in HepG2 and Bel_7402_ cells. We have also demonstrated that autophagy is a protective response that prevents matrine-treated HCC cells from undergoing apoptosis, and that the cytotoxic effect of matrine on hepatoma cells is mainly caused by apoptosis. Blockage of autophagic flux might promote cell-switching to apoptotic cell death. The combination of matrine and autophagic inhibitor CQ show a synergic effect on inducing apoptosis of human hepatoma cells.
